# Modern Fluorescence Strategies for Honey Characterization: Analytical Advances, Emerging Technologies, Methodological Challenges, and Future Perspectives

**DOI:** 10.3390/foods15071268

**Published:** 2026-04-07

**Authors:** Krastena Nikolova, Daniela Batovska, Galia Gentscheva, Tinko Eftimov, Yulian Tumbarski

**Affiliations:** 1 Department of Physics and Biophysics, Faculty of Pharmacy, Medical University of Varna, 84 Tzar Osvoboditel Blvd., 9000 Varna, Bulgaria; 2 Institute of Chemical Engineering, Bulgarian Academy of Sciences, Acad. G. Bonchev Str., Bl. 103, 1113 Sofia, Bulgaria; 3 Institute of General and Inorganic Chemistry, Bulgarian Academy of Sciences, Acad. G. Bontchev Str. Bl. 11, 1113 Sofia, Bulgaria; nisheva@svr.igic.bas.bg; 4 Centre de Recherche en Photonique, Université du Québec en Outaouais, 101 rue St-Jean Bosco, Gatineau, QC J8Y 3G5, Canada; tinko.eftimov@uqo.ca; 5 Central Laboratory for Applied Physics, Bulgarian Academy of Sciences, 4002 Plovdiv, Bulgaria; 6 Department of Microbiology and Biotechnology, Technological Faculty, University of Food Technologies, 4002 Plovdiv, Bulgaria; tumbarski@abv.bg

**Keywords:** adulteration detection, chemometrics, excitation–emission matrix, fluorescence spectroscopy, honey authentication

## Abstract

Honey authenticity control remains analytically challenging due to the complexity of its matrix and the increasing sophistication of adulteration practices. While chromatographic, spectrometric, and isotopic methods provide high confirmatory accuracy, their routine application is constrained by cost, time, and infrastructure requirements. In this context, fluorescence spectroscopy has emerged as a rapid, non-destructive, and cost-efficient screening approach capable of capturing subtle matrix-level compositional variations. This review critically evaluates the application of steady-state and excitation–emission matrix (EEM) fluorescence in honey quality and authenticity assessment. Fluorescence is positioned within tiered analytical frameworks as a first-line or intermediate screening tool preceding confirmatory chromatographic or NMR-based analyses. Emphasis is placed on intrinsic fluorophore domains, excitation–emission measurement strategies, and chemometric interpretation, including multiway analysis and supervised classification models. Recent developments in portable LED-based systems, laser-induced fluorescence, nanoparticle-based probes, and data-fusion strategies are discussed alongside key limitations related to matrix effects, spectral overlap, reproducibility, and model transferability. The review provides a structured framework for the strategic integration of fluorescence spectroscopy into contemporary honey authentication workflows.

## 1. Introduction

Honey is a high-value natural product characterized by a chemically complex composition and a biologically regulated production process [1,2]. Its premium market value, combined with sustained global demand, increases vulnerability to economically motivated adulteration and misrepresentation [3,4]. In addition to the deliberate addition of sugar syrups, modern adulteration practices increasingly involve technologically refined strategies designed to evade conventional quality control procedures [5,6]. Residues originating from agricultural and apicultural practices, including pesticides and antibiotics, further complicate authenticity assessment and safety evaluation [4,7]. Consequently, reliable analytical tools are essential for verifying honey quality, confirming botanical and geographical origin, and detecting deviations from genuine production [8,9,10,11].

Regulatory control is primarily based on the Codex Alimentarius Standard for Honey, which defines compositional parameters such as moisture content, sugar profile, electrical conductivity, acidity, and 5-hydroxymethylfurfural levels [12]. Although these criteria provide an important baseline for trade and safety, they rely mainly on bulk physicochemical indicators and may not reliably detect sophisticated adulteration practices or resolve complex questions related to botanical and geographical origin [7,13,14,15].

Advanced chromatographic and spectrometric techniques—including high-performance liquid chromatography (HPLC) [9], ultra-high-performance liquid chromatography (UHPLC) [16], liquid chromatography–high-resolution mass spectrometry (LC-HRMS) [10], liquid chromatography–tandem mass spectrometry (LC-MS/MS) [17], solid-phase microextraction–gas chromatography–mass spectrometry (SPME-GC-MS) [18], and nuclear magnetic resonance (NMR) spectroscopy [19]—provide high analytical accuracy and molecular specificity. Stable isotope ratio analysis (e.g., IRMS-based approaches) represents another powerful confirmatory technique widely applied in honey authentication, particularly for detecting C4 sugar adulteration and more subtle isotopic deviations. Recent developments have further extended its applicability through indirect detection strategies based on isotopic signatures of derivatized honey sugars [20]. However, the routine implementation of these techniques is often constrained by high operational costs, time-intensive workflows, extensive sample preparation, and the need for specialized infrastructure and expertise [8].

**Figure 1:**
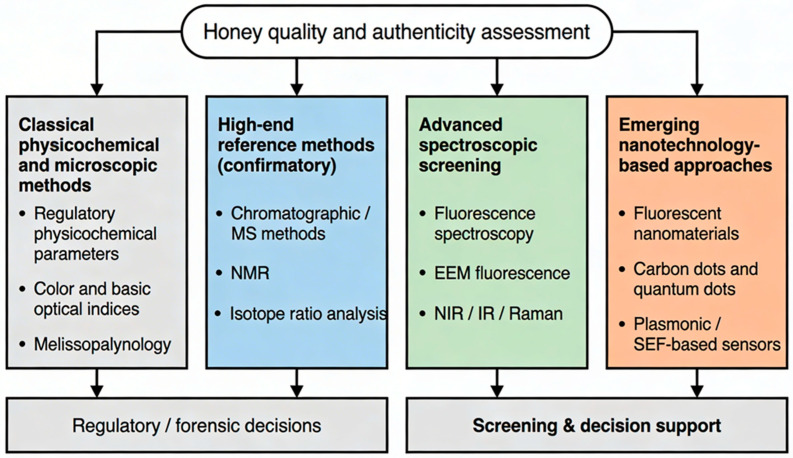
Schematic overview of analytical strategies for honey quality evaluation and authenticity assessment, including confirmatory techniques, spectroscopic screening approaches, and emerging nanotechnology-based sensing platforms.

These practical limitations have stimulated interest in rapid, non-destructive spectroscopic approaches. As illustrated in Figure 1, these methods can be positioned within tiered analytical workflows, where rapid screening techniques complement more advanced confirmatory analyses. Vibrational spectroscopic techniques, including near-infrared (NIR), Fourier-transform infrared (FTIR) [21], and Raman spectroscopy [22,23,24], have demonstrated reliable performance in detecting syrup adulteration and assessing botanical origin, particularly when combined with chemometric modeling [24,25,26,27]. Ultraviolet–visible (UV–Vis) absorption spectroscopy has also been applied for rapid compositional screening [6,13], although strong absorption in darker honey varieties may reduce analytical discrimination [28]. These constraints highlight the need for complementary optical approaches capable of resolving subtle compositional variations beyond absorption-based measurements.

Among optical techniques, fluorescence spectroscopy has attracted growing attention due to its high sensitivity to compositional changes within complex food matrices [29]. When combined with multivariate statistical tools such as principal component analysis (PCA), linear discriminant analysis (LDA), and partial least squares (PLS) regression, fluorescence measurements enable the discrimination of subtle spectral differences that may remain undetected by conventional bulk parameters [30,31,32]. Over the past decade, steady-state and excitation–emission matrix (EEM) fluorescence approaches have been increasingly applied to characterize monofloral honeys, differentiate botanical and geographical origin, and detect exogenous sugar syrups and other non-authentic additives [29,30,33].

Fluorescence spectroscopy is not intended to replace confirmatory techniques such as chromatography, mass spectrometry, or NMR, which remain essential for compound-level identification and quantitative validation [6,8]. Instead, fluorescence methods are more appropriately positioned as complementary tools within structured authentication workflows, where rapid screening can guide targeted confirmatory analysis.

Against this background, the present review critically evaluates fluorescence-based approaches for honey quality and authenticity assessment, focusing on intrinsic fluorophore domains, excitation–emission strategies, chemometric modeling, and methodological limitations. Particular attention is given to the positioning of fluorescence within tiered authentication systems.

This review aims to provide a structured analytical framework for integrating fluorescence spectroscopy into contemporary honey authentication strategies, clarifying both its analytical capabilities and its practical constraints. The major analytical approaches currently applied in honey classification and authenticity control are summarized in Figure 1. This provides a schematic overview of confirmatory techniques, spectroscopic screening methods, and emerging fluorescence-based strategies.

## 2. Fluorescence-Based Methodology for Honey Analysis

### 2.1. Honey as a Complex Fluorescent Matrix

Honey is a chemically and physically heterogeneous system in which fluorescence arises from the combined emission of multiple naturally occurring fluorophores embedded within a viscous and optically dense matrix. Its composition varies with botanical origin, environmental conditions, and post-harvest factors, leading to substantial inter-sample variability [34,35,36,37].

Fluorescence spectra of honey typically exhibit broad and strongly overlapping emission bands [29,34]. Spectral shifts are generally limited, and differences between samples are most often reflected in variations in fluorescence intensity rather than the appearance of distinct spectral features. This behavior has been consistently observed across steady-state, synchronous, and excitation–emission matrix (EEM) fluorescence studies [29,30,38].

Because multiple fluorophores contribute simultaneously to the observed signal, honey fluorescence is best interpreted as an integrated matrix fingerprint rather than a sum of independently resolvable components. Direct attribution of individual spectral features to specific molecular species is therefore generally not feasible, even when advanced acquisition strategies are applied [29,30,32,39].

The fluorescence response is further influenced by matrix-related optical effects. Inner-filter effects, self-absorption, light scattering, and fluorescence quenching are particularly pronounced in darker or highly concentrated honeys and may distort excitation–emission relationships, weakening the direct relationship between measured intensity and fluorophore concentration [40,41,42].

This intrinsic complexity explains why measurements acquired across multiple excitation–emission conditions, combined with multivariate data analysis, are essential for meaningful interpretation. Numerous authentication and adulteration studies have demonstrated that reliable discrimination is achievable only when the full multidimensional fluorescence response is evaluated using appropriate chemometric approaches [38,41,42].

### 2.2. Intrinsic Fluorophores Contributing to Honey Fluorescence

The fluorescence response of honey is determined by several classes of intrinsic fluorophores that differ in chemical structure, quantum yield, and excitation–emission behavior. These compounds originate from proteins, phenolic constituents, and reaction products formed during honey maturation and storage, each contributing to distinct spectral regions within the overall fluorescence profile [29,30,35].

Aromatic amino acids represent a major source of fluorescence, particularly within the protein-like spectral domain. Tryptophan typically provides the dominant contribution, whereas tyrosine and phenylalanine exhibit lower fluorescence intensity and play a secondary role. The overall influence of amino-acid-derived fluorescence varies among honey types and is associated with protein content and matrix composition [29,30].

Phenolic acids and flavonoids represent another major group of intrinsic fluorophores in honey. Their fluorescence reflects both the concentration and diversity of phenolic compounds, which are closely associated with floral origin and environmental conditions. Consequently, this group is frequently linked to botanical differentiation, although substantial spectral overlap among individual compounds limits direct molecular attribution [30,35,39,43,44].

Fluorescent compounds formed through Maillard reactions during honey maturation, storage, or thermal exposure also contribute significantly, particularly in darker honeys. These products arise from non-enzymatic reactions between sugars and amino components and are commonly associated with long-wavelength emission regions [29,37,40,41,45].

Additionally, less prominent fluorescence originates from vitamins (e.g., riboflavin), pigments, and other low-molecular-weight constituents. Although detectable under specific excitation conditions, their influence is generally subordinate to that of amino acids, phenolic compounds, and Maillard-derived products and rarely dominates the overall fluorescence response [32,35,46,47].

Because these fluorophore classes emit concurrently within overlapping spectral regions, individual fluorescence features cannot be uniquely assigned to specific molecular species. Instead, honey fluorescence spectra represent composite signals reflecting the combined behavior of multiple fluorophore populations rather than discrete chemical markers [30,42].

The principal intrinsic fluorophores contributing to honey fluorescence and their characteristic spectral regions are summarized in Table 1.

Although honey fluorescence represents a composite matrix signal characterized by overlapping emission features, the relative predominance of specific fluorescence domains may vary among botanical types under particular experimental conditions. Representative examples reported in the literature are summarized in Table 2.

As reflected in the fluorescence domains summarized in Table 1 and Table 2, the observed differences in fluorescence behavior among honey types arise from variations in the relative contributions of intrinsic fluorophore classes. In particular, phenolic compounds and flavonoids contribute predominantly to mid- and long-wavelength emissions, whereas protein-related fluorescence is associated with shorter wavelengths. In darker honeys, increased formation of Maillard reaction products enhances long-wavelength emission, while lighter honeys typically exhibit stronger protein- and phenolic-related fluorescence. As a result, the dominance of specific excitation–emission regions reflects the combined influence of botanical origin, compositional profile, and processing or storage conditions, rather than the presence of individual marker compounds.

### 2.3. Fluorescence Measurement Strategies in Honey Analysis

Fluorescence-based investigations of honey primarily employ two measurement approaches: steady-state fluorescence and excitation–emission matrix (EEM) fluorescence. These approaches differ in acquisition strategy, data dimensionality, and analytical capabilities [30,32]. Their principal characteristics are summarized in Table 3.

Steady-state fluorescence involves recording emission or excitation spectra at fixed wavelengths and is mainly suited for rapid screening and detection of pronounced compositional changes, such as those associated with adulteration or thermal treatment [29,30,51]. Because the measured signal reflects the combined emission of multiple fluorophores, steady-state spectra typically exhibit broad features with limited spectral specificity, which reduces their resolving power in complex classification tasks [29,30,52].

In contrast, EEM fluorescence records emission spectra across a range of excitation wavelengths, producing a three-dimensional (3D) dataset that describes the fluorescence response over a broad spectral region [29,39]. This multidimensional structure provides access to multiple excitation pathways within a single experiment and enhances sensitivity to compositional differences that may remain obscured under fixed-excitation conditions [30,41].

The higher informational content of EEM datasets is accompanied by increased dimensionality and strong inter-variable correlations resulting from spectral overlap and matrix-related optical effects [32,33,53]. Consequently, EEM fluorescence is inherently linked to multivariate data analysis, and chemometric methods are essential for meaningful interpretation [41,54].

Within this analytical framework, EEM fluorescence has become the preferred approach for applications requiring high discrimination power, including authenticity assessment, evaluation of storage- or heat-induced changes, and differentiation according to botanical or geographical origin [17,30,55,56,57,58].

In summary, steady-state fluorescence is a rapid and cost-effective method for preliminary screening, providing results within seconds; however, it exhibits low resolution due to the influence of overlapping signals within the complex matrix of honey. In contrast, excitation–emission matrix (EEM) fluorescence requires a higher investment in instrumentation and longer acquisition times (several minutes), but offers substantial potential for generating a detailed “fingerprint” that enables the determination of origin and authenticity.

The primary limitation of the steady-state method lies in its inaccuracy in detecting adulteration, whereas the main challenge associated with EEM fluorescence is the requirement for specialized software to process complex three-dimensional data. The fundamental differences between the two approaches lie in the excitation strategy, data dimensionality, and the analytical information obtained after measurement. While steady-state fluorescence relies on fixed excitation conditions and produces integrated emission spectra dominated by overlapping fluorophores, EEM fluorescence enables multidimensional characterization of the fluorescence profile by capturing multiple excitation pathways, thereby enhancing sensitivity to compositional variations within the matrix.

**Figure 2:**
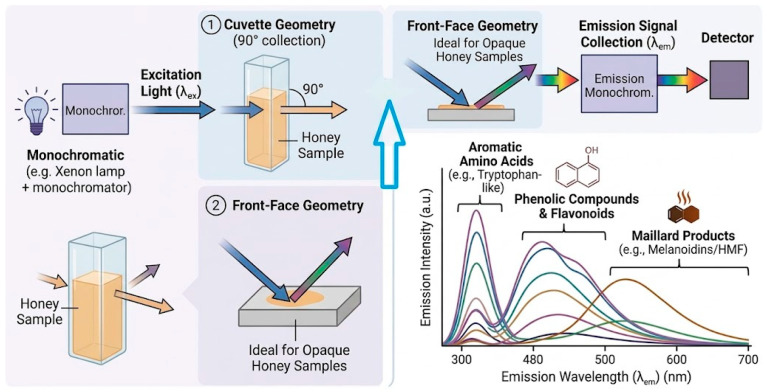
Steady-state fluorescence involves measurement of emission spectra at fixed excitation wavelength (s).

**Figure 3:**
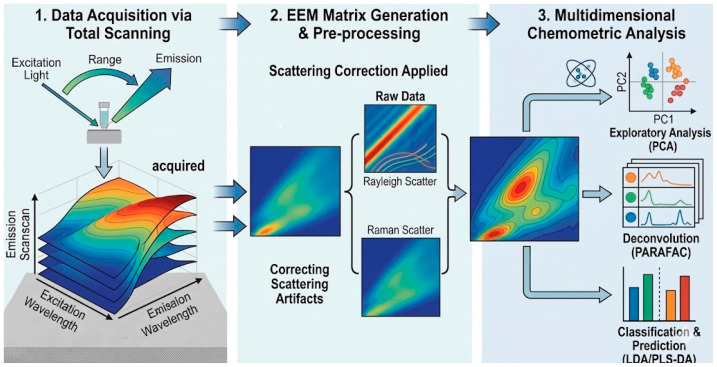
EEM fluorescence records emission spectra over a range of excitation wavelengths, generating a three-dimensional fluorescence dataset.

For better visualization of the differences between these two methods, Figure 2 and Figure 3 are presented, illustrating the principles of steady-state and EEM fluorescence based on the sources cited in this review [29,32,35,41,54].

### 2.4. Chemometric Interpretation of Honey Fluorescence Data

The intrinsic complexity of honey fluorescence data, resulting from overlapping emission features, matrix-related optical effects, and strong correlations among spectral variables, necessitates the use of chemometric methods for meaningful interpretation [8,29,32].

Unsupervised multivariate techniques, particularly principal component analysis (PCA), are commonly employed as an initial exploratory step. PCA enables visualization of sample clustering, detection of outliers, and identification of dominant sources of variance without prior class information. In honey fluorescence studies, this approach has been used to reveal differences associated with botanical origin, color, storage conditions, and adulteration level [30,47,52].

For EEM data, multiway chemometric approaches provide additional analytical advantages. Parallel factor analysis (PARAFAC) is especially suitable because it decomposes 3D fluorescence datasets into a limited number of mathematically independent components. In honey applications, PARAFAC has been used to resolve protein-like, phenolic-like, and Maillard-related fluorescence domains, thereby improving interpretability despite extensive spectral overlap [29,38,41,54].

Supervised classification and regression methods are applied when discrimination or quantitative prediction is required. Techniques such as linear discriminant analysis (LDA), partial least squares discriminant analysis (PLS-DA), and partial least squares regression (PLS) have been widely used to classify honey samples according to botanical or geographical origin, detect adulteration, and estimate the contribution of added sugar syrups [55,56,57]. In many studies, such models achieve classification accuracies exceeding 90% within specific datasets, although performance may decrease when applied to independent or more heterogeneous sample sets. However, these models are often dataset-dependent, and their performance may decrease when applied to independent or more heterogeneous sample sets, highlighting the importance of proper validation and model transferability. In addition to these approaches, class-modeling methods such as soft independent modeling of class analogy (SIMCA) have been employed for authenticity assessment, offering robust discrimination between authentic and adulterated samples while supporting simplified fluorescence-based screening strategies [59,60].

Machine-learning approaches have also been explored, particularly for EEM-based datasets. Convolutional neural networks and other deep-learning architectures have recently been applied to 3D fluorescence data for the authentication of monofloral honeys, demonstrating high classification accuracy under controlled conditions [61,62]. However, limited interpretability and the need for large training datasets currently restrict their routine application in authenticity-focused contexts, where transparency and chemical relevance remain essential [8,30].

### 2.5. Comparative Evaluation of Fluorescence Spectroscopy with Conventional Analytical Techniques

In order to define the position of fluorescence spectroscopy among modern and conventional strategies for honey evaluation, a critical comparison with established analytical techniques is required. Conventional chromatographic and chemical methods such as LC–MS/MS, GC–MS, and NMR provide high molecular specificity and quantitative accuracy; however, they are associated with high operational costs, complex sample preparation, and the need for specialized expertise.

In contrast, fluorescence spectroscopy is rapid, relatively inexpensive, and capable of detecting subtle variations in components related to storage conditions, adulteration, and botanical origin. Despite these advantages, it also has several limitations, primarily related to signals strongly affected by matrix effects, such as inner filter effects, quenching, and spectral overlap of components.

No analytical technique possesses exclusively advantages or disadvantages. Therefore, the most effective approach is the implementation of a multilevel analytical framework in which fluorescence spectroscopy functions as a first-line screening tool, guiding the selection of samples for subsequent confirmatory analysis using high-resolution techniques.

**Figure 4:**
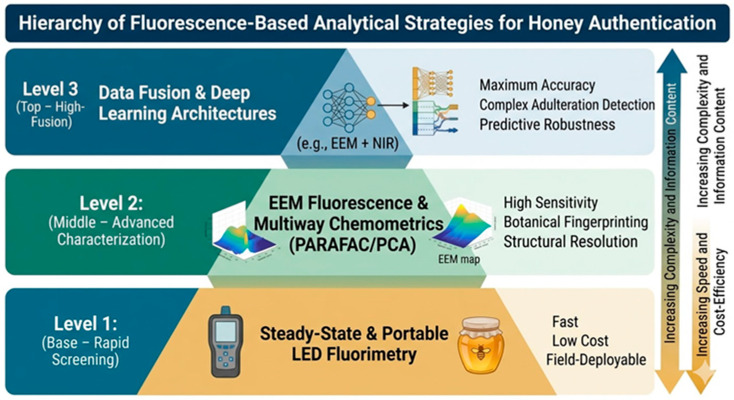
Hierarchical classification of fluorescence-based approaches for honey authenticity assessment.

For a clearer analytical comparison of the methods discussed, the evolution of analytical approaches—from rapid screening to detailed laboratory analysis—is illustrated in Figure 4.

## 3. Applications of Fluorescence Spectroscopy in Honey Analysis

The integration of fluorescence spectroscopy with chemometric analysis has enabled a range of practical applications in honey characterization and authenticity assessment [30,41].

### 3.1. Adulteration Detection

Detection of adulteration with low-cost sugar syrups represents one of the most extensive applications of fluorescence spectroscopy in honey analysis [55,57]. Changes in excitation–emission intensity patterns have been correlated with both the presence and level of added syrups, reflecting alterations in protein-related and carbohydrate-associated fluorescence domains [41,51,56]. When combined with multivariate classification or regression models, fluorescence-based approaches enable discrimination between authentic and adulterated honeys at concentration levels relevant for screening purposes [30,56]. Detection limits reported in the literature often fall within the range of approximately 5–20% added sugar syrup, although lower or higher values have also been reported depending on the honey type, matrix composition, and the chemometric approach applied [56]. Portable light-emitting diode (LED)-based systems coupled with non-targeted chemometric modeling, including SIMCA, have demonstrated effectiveness for both detection and quantification of adulteration, even in complex matrices such as stingless bee honey. These findings support the feasibility of simplified, field-deployable fluorescence screening tools [33,59,60,63].

Recent studies have further combined 3D fluorescence datasets with advanced chemometric and deep-learning strategies, confirming the potential of fluorescence-based methods to differentiate honey from syrup adulterants under controlled experimental conditions [57,61]. Reported classification accuracies for fluorescence-based models typically range between approximately 85% and 98% under controlled conditions, depending on the honey type, adulterant, and chemometric approach used.

### 3.2. Storage and Thermal Effects

Beyond adulteration detection, fluorescence fingerprints have also been associated with storage duration and thermal treatment. Such changes are typically manifested as increased long-wavelength emission linked to the formation of Maillard reaction products [30,64]. Reported fluorescence responses generally show consistent intensity increases in this spectral region, although quantitative thresholds vary between studies depending on experimental conditions. In this context, laser-induced fluorescence approaches exploiting secondary inner-filter effects have been proposed for optical assessment of honey freshness through indirect monitoring of Maillard-related markers such as furosine [40,45].

### 3.3. Botanical and Geographical Differentiation

Fluorescence-based differentiation according to botanical and geographical origin remains more challenging compared to adulteration detection. When sufficiently large datasets and appropriate reference samples are available, multivariate analysis of excitation–emission matrix data has demonstrated promising classification performance [30,41]. Reported classification accuracies for botanical differentiation are generally lower and more variable than for adulteration detection, often reflecting dataset-specific performance. Reliable discrimination is achieved most effectively when full excitation–emission distributions are evaluated rather than isolated spectral features [30,41].

### 3.4. General Quality Monitoring

Beyond authentication, fluorescence methods have been explored for general quality monitoring, including identification of atypical samples and detection of processing-related alterations. In these applications, fluorescence functions as an integrative analytical tool that reflects collective compositional changes rather than individual marker compounds [30,64].

Overall, across these application domains, the practical value of fluorescence-based methods lies in their role within tiered analytical workflows. Although they do not replace confirmatory techniques such as chromatography or isotope-ratio analysis, fluorescence measurements provide a rapid and cost-effective screening layer that can substantially reduce analytical burden in routine honey authentication and quality control [8,30].

The major application areas, methodological requirements, and limitations discussed in this section are summarized in Table 4.

## 4. Emerging Fluorescence Technologies and Instrumental Innovations

Recent developments in fluorescence spectroscopy have expanded its analytical capabilities in honey research, driven by advances in instrumentation, chemometric methodologies, and sensor design. The principal innovation directions and their analytical characteristics are summarized in Table 5. These approaches differ not only in analytical performance but also in their level of maturity, practical feasibility, and requirements for data processing and standardization.

Among advanced analytical approaches, the combination of EEM fluorescence with multiway chemometric analysis remains particularly important for complex matrices such as honey. As outlined in Section 2.4, 3D fluorescence datasets provide detailed spectral fingerprints that are highly sensitive to compositional variation. However, extensive spectral overlap and matrix-related optical effects complicate direct interpretation, necessitating decomposition methods such as PARAFAC to resolve independent fluorescence components [54]. Despite its analytical power, this approach requires careful preprocessing, including correction for Rayleigh and Raman scattering, which may limit its use in real-time or simplified analytical environments. Compared to simpler fluorescence approaches, this strategy offers superior analytical resolution but requires more complex instrumentation and data processing.

Integration of complementary analytical modalities, commonly referred to as data fusion, represents another important development. Combining fluorescence measurements with techniques such as NIR spectroscopy, Raman spectroscopy, or electronic tongue systems enables more comprehensive characterization of honey by capturing both molecular-level and bulk compositional information [65,66]. Such multimodal strategies have demonstrated improved classification accuracy compared with single-method approaches, particularly for complex adulteration scenarios. However, these approaches involve increased experimental complexity and require the integration of multiple analytical platforms. In addition, the need for multiple instruments and data integration workflows may increase operational costs and limit routine implementation in standard analytical settings.

Technological miniaturization has enabled the development of portable fluorescence devices based on LED excitation sources. These systems offer narrow spectral bandwidth, low power consumption, reduced cost, and extended operational lifetime, making them suitable for field deployment and on-site screening applications [33,60]. Although portable devices typically provide lower spectral resolution than laboratory instruments, they represent a promising step toward decentralized honey quality monitoring. In parallel with these developments, fluorescence imaging and hyperspectral approaches are emerging as complementary tools, enabling spatially resolved fluorescence analysis and detection of sample heterogeneity [41]. Although still at an early stage of application in honey, these methods provide additional analytical dimensions for rapid and non-destructive screening.

Nanomaterial-based fluorescent probes represent an emerging direction with potential for selective detection of contaminants in honey. Carbon quantum dots (CQDs), metal–organic frameworks (MOFs), and related nanosystems enable analyte-responsive fluorescence modulation through mechanisms such as fluorescence resonance energy transfer (FRET), photoinduced electron transfer (PET), and inner-filter effects [67]. Antibiotic residues are of particular concern because treatment of bee colonies with tetracyclines and aminoglycosides may lead to accumulation in honey. CQD-based sensors, including heteroatom-doped variants, have demonstrated high sensitivity for tetracycline detection, while fluorescence quenching strategies have been applied to antibiotics such as ampicillin. More complex nanosystems incorporating MOFs and semiconductor materials have further extended detection capability to aminoglycosides including kanamycin and neomycin [68,69,70,71]. However, the practical implementation of such nanosystems remains challenging, as their synthesis and functionalization may involve significant cost and reproducibility issues; the lack of standardized protocols limits their transfer to routine analytical applications.

**Table 5:** Evolution of fluorescence spectroscopy in honey analysis: emerging directions, analytical scope, and limitations.

Innovation Direction	Core Principle	Analytical Scope	Level of Maturity	Main Advantages	Key Limitations
EEM + PARAFAC/Multiway Analysis	Decomposition of 3D excitation–emission matrices into independent components	Botanical and geographical differentiation; adulteration detection; storage and thermal effects	High (well-established in research)	Improved interpretability; resolution of overlapping fluorescence domains; robust classification performance	Requires advanced chemometric expertise; sensitive to preprocessing and model selection
Synchronous Fluorescence (SFS)	Simultaneous scanning of excitation and emission wavelengths with a constant wavelength offset (Δλ)	Rapid screening; improved resolution of overlapping fluorophores; preliminary discrimination of honey types and adulteration	Moderate	Reduced spectral complexity; enhanced selectivity compared to steady-state fluorescence; faster data acquisition	Lower information content than EEM; sensitivity to Δλ selection; limited discrimination in complex matrices
Data Fusion (EEM + NIR/Raman/electronic tongue)	Integration of complementary spectral or sensor datasets	Complex adulteration scenarios; improved origin discrimination	Moderate–High	Enhanced predictive robustness; reduced method-specific bias	Increased computational complexity; multi-instrument requirements
Portable LED-Based Fluorimetry	Miniaturized excitation sources with simplified spectral acquisition	Rapid screening; field deployment	Moderate	Low cost; fast analysis; suitable for decentralized testing	Reduced spectral resolution; limited compositional depth
Fluorescence Imaging/Hyperspectral Approaches	Spatially resolved fluorescence acquisition combined with spectral analysis across multiple wavelengths	Detection of sample heterogeneity; surface-level authentication; imaging-assisted classification and rapid screening	Emerging	Provides combined spatial and spectral information; non-destructive; suitable for rapid and visual screening	High data complexity; limited penetration depth; requires advanced data processing and instrumentation
Nanoparticle-Based Fluorescent Probes (CQDs, MOFs, aptasensors)	Target-specific fluorescence modulation (FRET, PET, IFE)	Detection of antibiotics, pesticides, heavy metals	Emerging (mainly proof-of-concept)	High sensitivity; analyte-specific response	Matrix interference; limited validation in real samples; regulatory uncertainty
Deep Learning on EEM Data	Nonlinear modeling of high-dimensional fluorescence datasets (e.g., CNN-based analysis of EEM data)	Automated authenticity classification	Emerging	High classification accuracy under controlled conditions	Limited interpretability; large training datasets required
Time-Resolved Fluorescence	Measurement of fluorescence lifetime instead of intensity	Potential separation of overlapping signals; improved selectivity	Early stage in honey analysis	Reduced dependence on intensity variations; enhanced discrimination potential	Requires specialized instrumentation; limited application studies in honey analysis

Note: Representative sources include [28,35,56,60,61,72], and related studies cited in the main text of Section 4.

Despite these promising analytical performances, matrix interference, limited validation in real samples, and regulatory uncertainties currently restrict routine implementation of nanosensor-based fluorescence methods in honey quality control.

## 5. Limitations and Future Perspectives

### 5.1. Methodological and Analytical Limitations

Despite the growing interest in fluorescence spectroscopy as a rapid and non-destructive analytical tool for honey analysis, several methodological and analyticial limitations must be acknowledged to ensure realistic interpretation of results and appropriate positioning of the technique within analytical workflows [14,30].

A primary constraint arises from matrix-related effects inherent to the physicochemical complexity of honey. Variations in color intensity, moisture content, viscosity, and crystallization state can significantly influence fluorescence signals through inner-filter effects, self-absorption, light scattering, and quenching phenomena [30,32]. Consequently, apparent excitation–emission patterns may reflect both compositional and optical properties, complicating direct comparison between honey types and across independent studies unless appropriate correction and normalization strategies are applied [30].

Sensitivity and detection limits represent an additional analytical limitation, particularly in adulteration analysis. Although fluorescence-based approaches are effective for rapid screening of common sugar adulterants, reliable detection is generally restricted to moderate or higher adulteration levels and remains dependent on honey composition and baseline fluorescence characteristics [41,56,73].

Methodological variability further constrains reproducibility. Differences in excitation–emission ranges, spectral resolution, sample dilution protocols, measurement geometry, and preprocessing procedures can lead to substantial discrepancies between datasets [30,32]. In the absence of harmonized acquisition and correction protocols, inter-laboratory transferability remains limited, particularly for excitation–emission matrix (EEM) datasets [30].

Finally, the strong reliance on multivariate and machine-learning models introduces additional analytical complexity. Model performance is frequently dataset-specific and dependent on training-set representativeness, while limited generalization and interpretability may restrict application in regulatory or forensic contexts [8,30].

A major obstacle to the widespread application of fluorescence methods is the lack of standardization. Differences in instrument settings, sample preparation, and data processing lead to different results and make comparison between individual laboratories difficult. This reduces the trust in these models, especially when used for authenticity control and regulatory purposes.

The main sources of variability of the fluorescent signals emitted by honey samples are summarized in Table 6 according to scientific sources cited in the text [29,30,32,38,41,54].

### 5.2. Strategic Integration Within Tiered Authentication Workflows

Despite these methodological constraints, the operational characteristics of fluorescence spectroscopy support its strategic integration within tiered authentication workflows. In most routine applications, sample preparation is limited to simple dilution without extraction or derivatization, thereby reducing procedural complexity and potential sources of variability. Spectral acquisition is rapid, non-destructive, and solvent-free, enabling high-throughput screening under routine laboratory conditions [29].

These features position fluorescence spectroscopy as a preliminary screening layer capable of prioritizing samples for subsequent confirmation by reference techniques such as chromatography, isotope-ratio analysis, or nuclear magnetic resonance (NMR) spectroscopy [8,30]. Rather than replacing confirmatory methods, fluorescence measurements can reduce analytical burden and improve workflow efficiency in large-scale authenticity monitoring.

The development of compact LED-based fluorimetric systems further supports decentralized or field-oriented screening scenarios [33]. However, effective implementation requires clearly defined decision thresholds, validated chemometric models, and appropriate quality-control procedures to prevent overinterpretation of screening-level outcomes.

Accordingly, the principal value of fluorescence spectroscopy in honey analysis lies not in compound-specific identification but in its capacity to provide rapid, integrative compositional fingerprints that guide targeted follow-up investigation.

### 5.3. Future Directions and Emerging Technologies

Future progress in fluorescence-based honey analysis will depend primarily on methodological harmonization and improved data robustness. The establishment of standardized acquisition protocols—including controlled dilution procedures, correction for inner-filter effects, and consistent excitation–emission ranges—will be essential to enhance inter-laboratory comparability and regulatory credibility [14,30]. The urgent need for standardized analytical protocols in honey analysis represents a critical bottleneck, as the lack of uniform measurement procedures currently hinders the collection of data covering diverse botanical and geographical origins.

Advances in multimodal data integration represent another important direction. Data-fusion strategies combining excitation–emission matrix fluorescence with complementary spectroscopic techniques, such as near-infrared or Raman spectroscopy, have demonstrated improved predictive stability and robustness in related food authenticity applications [28,61]. Such integrative approaches may mitigate dataset-specific bias while reducing reliance on increasingly complex, less interpretable machine-learning models.

In parallel, engineered fluorescent nanomaterials—including carbon quantum dots, aptamer-functionalized nanoparticle systems, and metal–organic framework-based emitters—are emerging as targeted extensions of fluorescence-based sensing. Unlike intrinsic matrix fluorescence, these probe-assisted platforms are designed for selective detection of specific residues, such as antibiotics or pesticides, potentially expanding the analytical scope of optical screening strategies in honey and other bee products. However, most reported systems remain at a proof-of-concept stage, and their routine implementation is limited by matrix complexity, validation gaps, and regulatory uncertainty. Consequently, nanoparticle-assisted fluorescence sensing should be regarded as a complementary tool rather than a replacement for established confirmatory methods

Overall, the evolution of fluorescence-based honey analysis is likely to follow a balanced trajectory: strengthening the robustness and standardization of intrinsic fluorescence fingerprinting while selectively integrating multimodal and probe-based technologies within structured, tiered analytical frameworks. Within such systems, fluorescence spectroscopy will continue to function as a rapid, integrative screening layer that supports efficient and scientifically grounded honey authentication workflows.

In this context, the present review highlights fluorescence spectroscopy as a rapid, cost-efficient, and accessible screening approach suitable for early-stage assessment, with results that can be further validated by centralized confirmatory analyses. By systematically summarizing fluorescence acquisition strategies—from steady-state and excitation–emission matrix measurements to portable and field-deployable fluorescence systems—and by clarifying the role of multivariate and supervised classification techniques, this review provides a practical methodological framework for first-line honey authenticity assessment. Such fluorescence-based screening tools are particularly suited for deployment at the producer level, in regional laboratories, or within preliminary control workflows, where they may support early detection of anomalies and guide the targeted use of accredited reference methods.

## 6. Concluding Remarks

The future development of fluorescence spectroscopy must progress from the stage of experimental validation toward full methodological standardization and implementation within multi-tier analytical systems. A primary priority is overcoming technical limitations associated with matrix effects and spectral overlap through the development of unified protocols for inner filter effect correction and the standardization of measurements across different instrumental platforms.

The next generation of analytical tools will rely on the integration of artificial intelligence and deep machine learning (Deep Learning) to process complex three-dimensional excitation–emission matrices (EEMs) with greater accuracy than traditional chemometric approaches. A critical step forward is the establishment of global, open-access digital libraries containing fluorescence fingerprints of authentic honey, which would serve as reliable references for the detection of emerging, high-technology forms of adulteration.

In parallel, technological efforts should focus on the miniaturization of optical components and the development of high-resolution portable sensors capable of enabling precise quality control outside laboratory environments. In this way, fluorescence spectroscopy will evolve into a scalable operational screening tool that effectively identifies anomalies in real time and optimizes the use of costly confirmatory analyses such as NMR and IRMS.

Within such a balanced framework, fluorescence spectroscopy should not be regarded as a substitute for established methods, but rather as a strategically positioned component of contemporary honey authentication strategies.

## Figures and Tables

**Table 1:** Principal intrinsic fluorophores contributing to honey fluorescence and their characteristic excitation–emission regions.

Fluorophore Class	Representative Compounds	Typical Excitation (nm)	Typical Emission (nm)	Analytical Relevance
Aromatic amino acids	Tryptophan, tyrosine, phenylalanine	270–295	330–360	Protein-related fluorescence; indicators of freshness, dilution, and adulteration
Phenolic acids	Caffeic acid, ferulic acid, *p*-coumaric acid, ellagic acid	300–350	400–500	Botanical influence; mid-wavelength fluorescence characteristics
Flavonoids	Quercetin, kaempferol, hesperetin	350–380	450–550	Floral source contribution; antioxidant-associated fluorescence
Maillard products	Advanced glycation end products, melanoidin-like structures, melanoidins	350–450	>500	Indicators of storage, aging, and thermal treatment
Vitamins	Riboflavin and related products	~370	~520	Minor contributors; condition-dependent fluorescence

Note: 5-Hydroxymethylfurfural, although an important marker of honey aging and thermal processing, exhibits weak fluorescence and is typically quantified using complementary analytical methods rather than fluorescence spectroscopy.

**Table 2:** Reported predominant fluorescence domains in selected botanical honeys (representative literature examples).

Botanical Type of Honey	Reported Dominant Excitation–Emission Regions (λex/λem, nm)	Predominant Fluorescence Domains (Matrix-Level Interpretation)	References
Acacia (*Robinia pseudoacacia* L.)	280–350/340–520	Relative predominance of protein-like and phenolic-like domains; typically associated with lighter-colored matrices	[48]
Linden (*Tilia* spp.)	~370/~550	Enhanced long-wavelength emission attributed to increased contribution of Maillard-related components	[27]
Sunflower (*Helianthus annuus* L.)	~320/~450	Broad phenolic-like emission region with substantial overlap with other floral honeys	[27]
Chestnut (*Castanea sativa* Mill.)	~380/~480	Increased mid- to long-wavelength emission consistent with darker matrix composition and higher phenolic content	[48]
Honeydew honey	~270/~300	Elevated short-wavelength (protein-like) fluorescence relative to floral honeys within studied datasets	[49]
Polyfloral honey	370–500/400–550	Strongly overlapping fluorescence domains reflecting mixed botanical origin and composite fluorophore contributions	[29]
Manuka (*Leptospermum scoparium J.R.Forst. & G.Forst.*)	270/365 and 330/470	Predominant domains associated with methylglyoxal-related and phenolic components in specific datasets	[50]
Kanuka (*Kunzea ericoides (A.Rich.) Joy Thomps.*)	275/305 and 445/525	Similar domain distribution to Manuka but with lower intensity in the methylglyoxal-associated region	[50]

Note: Reported excitation–emission regions are derived from individual studies and depend on instrumentation, dilution procedures, spectral resolution, and measurement geometry. These values represent dominant fluorescence domains observed under specific experimental conditions and should not be interpreted as universal or unique botanical markers.

**Table 3:** Comparison of steady-state and excitation–emission matrix fluorescence approaches in honey analysis.

Feature	Steady-State Fluorescence	EEM Fluorescence
Excitation strategy	Fixed excitation wavelength(s)	Systematic scanning of excitation wavelengths
Emission acquisition	Emission spectrum at fixed excitation	Emission spectra recorded for each excitation wavelength
Data dimensionality	2D (intensity vs. wavelength)	3D (intensity vs. excitation × emission)
Typical data output	Single emission or excitation spectrum	Fluorescence matrix/landscape/fingerprint
Representative methods	Conventional emission fluorescence; excitation spectra; synchronous fluorescence; front-face fluorescence	Excitation–emission matrix fluorescence; total luminescence spectroscopy
Information content	Limited, dominated by overlapping fluorophore signals	High, captures multiple excitation pathways simultaneously
Sensitivity to subtle differences	Moderate	High
Susceptibility to spectral overlap	High	High, but addressable by multivariate analysis
Need for chemometrics	Optional	Essential
Common analytical applications	Rapid screening; detection of gross adulteration or thermal effects	Authentication; botanical and geographical differentiation; multivariate classification
Typical chemometric tools	PCA, PLS (optional)	PARAFAC, PCA, PLS, multiway analysis

**Table 4:** Summary of major application areas of fluorescence spectroscopy in honey analysis, including analytical scope, methodological requirements, and key limitations.

Application Area	What Fluorescence Captures	Methodological Note	Limitations
Adulteration detection	Changes in intensity patterns; protein- and Maillard-related domains	EEM combined with chemometrics required	Detection limits; honey-type dependence
Botanical differentiation	Composite fluorescence fingerprints	Region- and dataset-specific models	Requires reference libraries
Geographical origin	Indirect spectral patterns; no unique markers	Limited model transferability	High risk of overinterpretation
Storage/heat treatment	Increase in long-wavelength emission	Sensitive to Maillard-related changes	Confounded by natural color variation
Field deployability	Rapid, non-destructive measurements	LED-based systems feasible	Reduced spectral resolution

Abbreviations: EEM, excitation–emission matrix; LED, light-emitting diode.

**Table 6:** Sources of variability in the fluorescence signal from bee pollen and possibilities for their resolution.

Source of Variability	Description	Analytical Impact	Solution Approaches
Excitation–emission settings	Differences in the ranges λ_ex_/λ_em_, pitch, gap width	Changes the shape of the spectrum and the intensity distribution	Standardized measurement ranges and resolution
Sample preparation	Differences in dilution, solvent, filtration	Affects the intra-filter effect and fluorescence intensity	Defined protocols for dilution and optical density control
Measurement geometry	Right-angle and front-face configurations	Affects scattering and absorption effects	Calibration and reporting according to geometry
Instrumental differences	Detector sensitivity, light source type (lamp vs. LED)	Limits comparability between devices	Validation and cross-calibration of instruments
Internal filter effect	Absorption of excitation/emission light in the sample	Distorts intensity dependencies	Mathematical correction and controlled dilution
Scattering (Rayleigh/Raman)	Elastic and inelastic scattering	Introduces artifacts into EEM data	Correction algorithms
Preliminary data processing	Baseline correction, normalization, smoothing	Strongly affects chemometric results	Use of standardized processing protocols
Chemometric modeling	Choice of PCA, PLS, PARAFAC, ML models	Interpretation and classification depend on the model	External validation and transparent reporting
Sample heterogeneity	Botanical origin, color, viscosity	High natural variability in fluorescence	Use of representative reference sets

## Data Availability

The original contributions presented in this study are included in the article. Further inquiries can be directed to the corresponding authors.

## Abbreviations

The following abbreviations are used in this manuscript:

EEM	Excitation–emission matrix
HPLC	High-performance liquid chromatography
UHPLC	Ultra-high-performance liquid chromatography
LC-HRMS	Liquid chromatography-high resolution mass spectrometry
LC-MS/MS	Liquid chromatography–tandem mass spectrometry
GC-MS	Gas chromatography–mass spectrometry
SPME-GC-MS	Solid-phase microextraction-gas chromatography-mass spectrometry
NMR	Nuclear magnetic resonance
UV-Vis	Ultraviolet–visible
NIR	Near-infrared spectroscopy
IR/FTIR	Fourier-transform infrared spectroscopy
PCA	Principal component analysis
LDA	Linear discriminant analysis
PLS	Partial least squares
PARAFAC	Parallel factor analysis
LED	Light-emitting diode
SIMCA	Soft independent modelling of class analogy
CQDs	Carbon quantum dots
FRET	Resonance energy transfer
PET	Photoinduced electron transfer
IFE	Inner filter effect
MOFs	Metal–organic frameworks
SDZ	Sulfadiazine
LOD	Limit of detection
MTZ	Metronidazole
